# Post cataract surgery refractive surprise due to intraocular lens mislabeling

**DOI:** 10.1002/ccr3.9104

**Published:** 2024-06-21

**Authors:** Saeed Shokoohi Rad, Mohammad Reza Ansari Astaneh, Mohammad Yaser Kiarudi, Seyed Hossein Ghavami Shahri, Hamid Reza Heidarzadeh

**Affiliations:** ^1^ Eye Research Center Mashhad University of Medical Sciences Mashhad Iran

**Keywords:** cataract, intraocular lens, phacoemulsification, refractive surprise

## Abstract

**Key Clinical Message:**

If an intraocular lens (IOL) is incorrectly labeled, problems can arise for surgeons and patients. Precise biometry is important to accurately determine the IOL power and prevent the implantation of the wrong IOL. Labeling and verifying IOLs with accuracy is crucial to ensuring the best possible results of cataract surgery.

**Abstract:**

Mislabeling of IOLs can cause unpredictable problems for surgeons. However, we can prevent incorrect IOLs by using precise biometry to determine accurate IOL power and safely implant the correct IOL. A 50‐year‐old female with no medical or ocular history came to our clinic complaining of decreased vision in both eyes that had been ongoing for several months. After being diagnosed with cataracts, primary angle closure suspect, and high hyperopia, the patient underwent phacoemulsification surgery. A posterior chamber IOL was implanted, and visco‐goniosynechialysis was performed. During follow‐up appointments, it was discovered that the patient had an uncorrected visual acuity of 20/50 in her right eye, which was corrected to 20/20 with a + 7.00 D lens. Upon further evaluation, it was determined that the source of the error was due to a manufacturing mislabeling of the IOL power. The patient then underwent successful IOL exchange surgery, and her best‐corrected visual acuity became 20/20 with no significant refraction. This case highlights an uncommon source of refractive surprise after phacoemulsification surgery, successfully managed with IOL exchange surgery using the same IOL power from a different brand.

## 1 INTRODUCTION

An excellent visual outcome without the need for spectacles is expected after uneventful phacoemulsification surgery in modern ophthalmology. This goal can be achieved by ensuring accurate biometry, selecting the appropriate intraocular lens (IOL) power formula, and using modern cataract extraction techniques such as phacoemulsification with foldable monofocal, multifocal, toric, or extended depth of focus IOLs. Errors in biometry, such as inaccurate measurements of axial length and keratometry, IOL calculation formula, IOL insertion, and lens constant errors, are the primary sources of post‐cataract surgery refractive surprises.^1^

Other common reasons for incorrect IOL implantation include selecting the wrong IOL, errors in transcribing or interpreting handwritten notes, changes in the order of the surgical list, confusion between the right and left eye, issues with patient identification, misfiling of biometric data, incorrect IOL information written on the theater whiteboard, unavailability of the optimal IOL power in stock, implanting the wrong IOL power after a complicated surgery, mistakes in patient notes, communication errors, and, in rare cases, a mislabeled IOL by the manufacturer.^2^

The present case report describes a patient who exhibited refractive surprise after an uneventful phacoemulsification surgery, chiefly due to an IOL that had been mislabeled. The patient subsequently underwent an IOL exchange surgery, which resulted in a favorable outcome of 20/20 uncorrected visual acuity (UCVA).

## 2 CASE HISTORY/EXAMINATION

A woman, aged 50, with no prior medical or ocular history, came to our clinic complaining of a decrease in vision in both eyes that had been ongoing for several months. Upon examination, it was found that her best‐corrected visual acuity (BCVA) was 20/28 in the right eye with a + 5.5 D sphere, and 20/25 in the left eye with a + 4 D sphere. The slit‐lamp examination showed cataracts in both eyes, while the rest of the examination was normal. Intraocular pressure (IOP) was measured using Goldmann applanation tonometry and was found to be 22 mmHg in the right eye and 18 mmHg in the left eye. Gonioscopy was performed using Volk 4 mirror gonio‐lens, and the angle was occluded without any peripheral anterior synechiae in both eyes. Fundus examination showed a 0.5 cup‐disc ratio in both eyes, with the rest of the examination being normal.

## 3 METHODS

**FIGURE 1:**
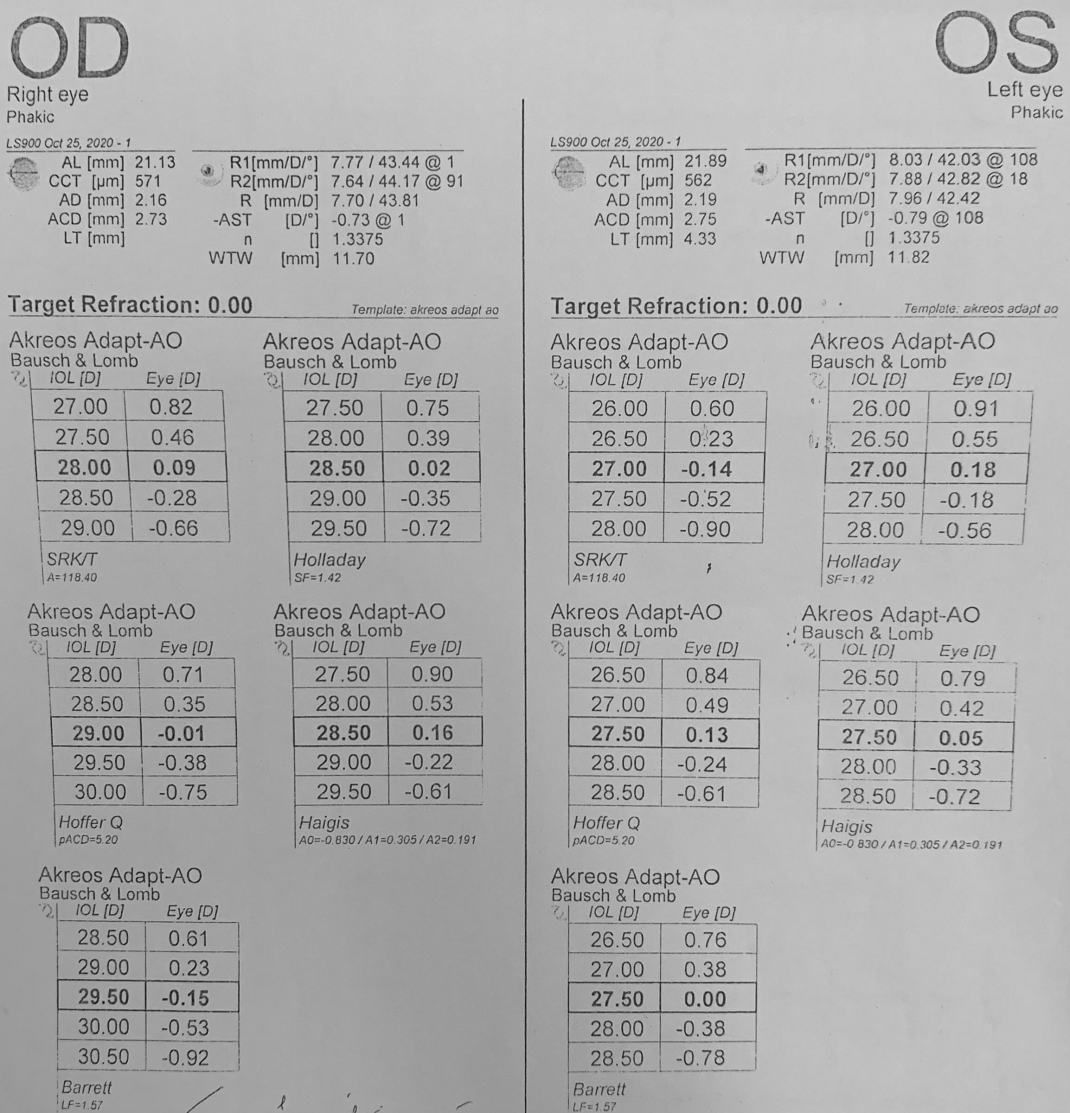
Patient phakic IOL power calculation printout (Haag Streit LENSTAR® EyeSuiteTM IOL V4.2.1).

The patient was diagnosed with cataract, primary angle closure suspect, and high hyperopia. To treat this, we planned phacoemulsification with posterior chamber IOL implantation and viscogoniosynechialysis for the right eye. We used the Haag Streit LENSTAR® EyeSuiteTM IOL V4.2.1 to calculate the IOL power, with an Akreos® Adapt‐AO IOL A constant of 118.4. According to the Barrett Universal II formula, the proper IOL power for the patient was +29.5D (Figure 1). As the patient's axial length was 21.13 mm, we double‐checked the IOL power with the Hoffer Q formula, which confirmed our selection. The surgery was successful without complications, and we implanted a+29.5D Akreos® Adapt‐AO monofocal hydrophobic acrylic one‐piece IOL (BAUSCH & LOMB).

**FIGURE 2:**
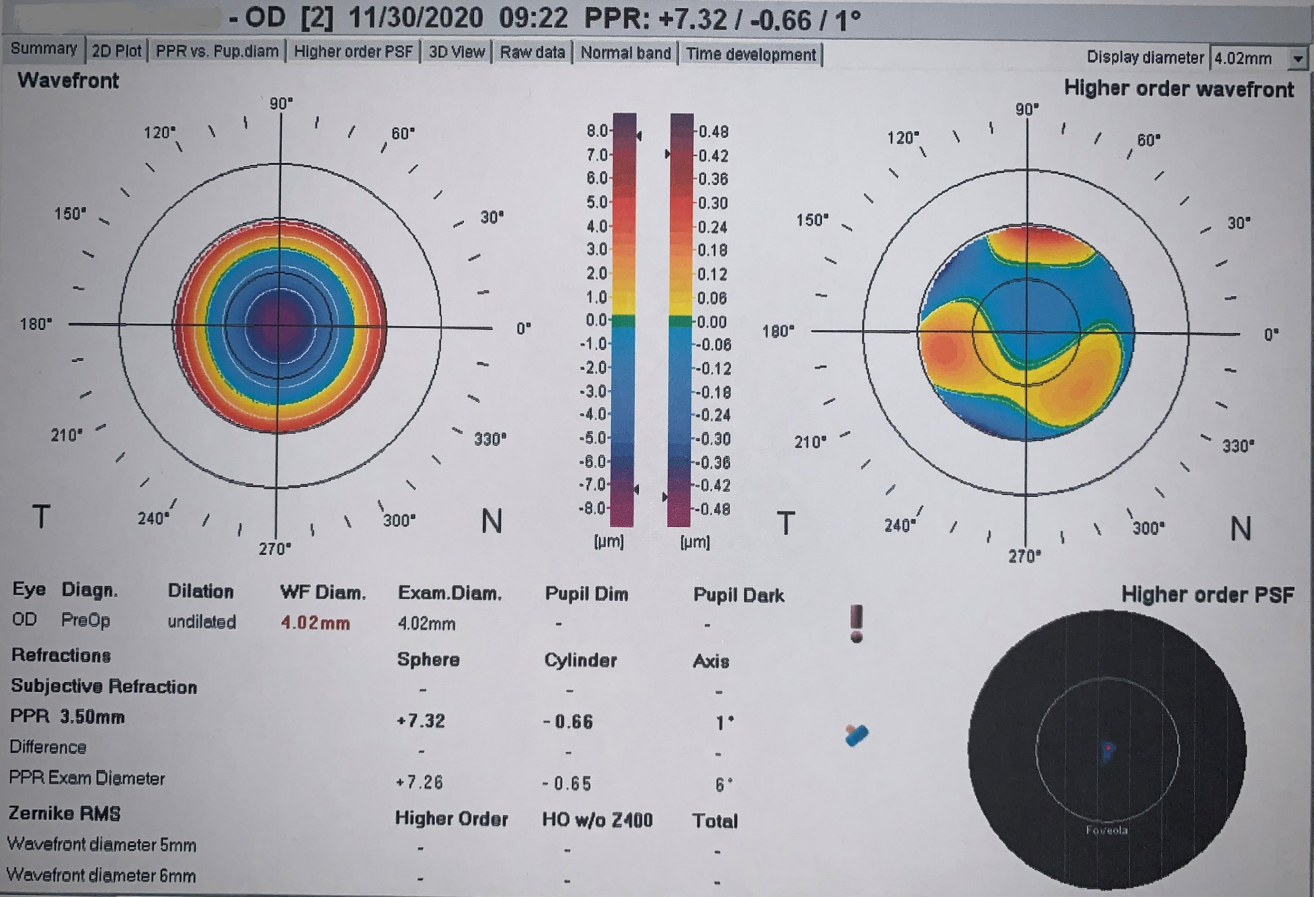
Aberrometry printout disclosed the same refraction as the subjective refraction (ZyWave 5.2, Technolas GmbH).

Five days after the surgery, during a follow‐up, the patient had a 20/50 uncorrected visual acuity (UCVA) for her right eye. Since the surgery had gone smoothly and the patient's postoperative condition was stable, the cause for the drop in UCVA was unclear. We performed a refraction evaluation with autorefractor and found that her vision improved to 20/20 with a+7.00 D refraction. An aberrometry image disclosed a similar refraction as autorefraction (Figure 2). We repeated the biometry and IOL calculations three times, and all of them were similar to the preoperative calculations. It is important to note that the axial length measurement in the right eye was corrected for the presence of an IOL.

## 4 CONCLUSION AND RESULTS

**FIGURE 3:**
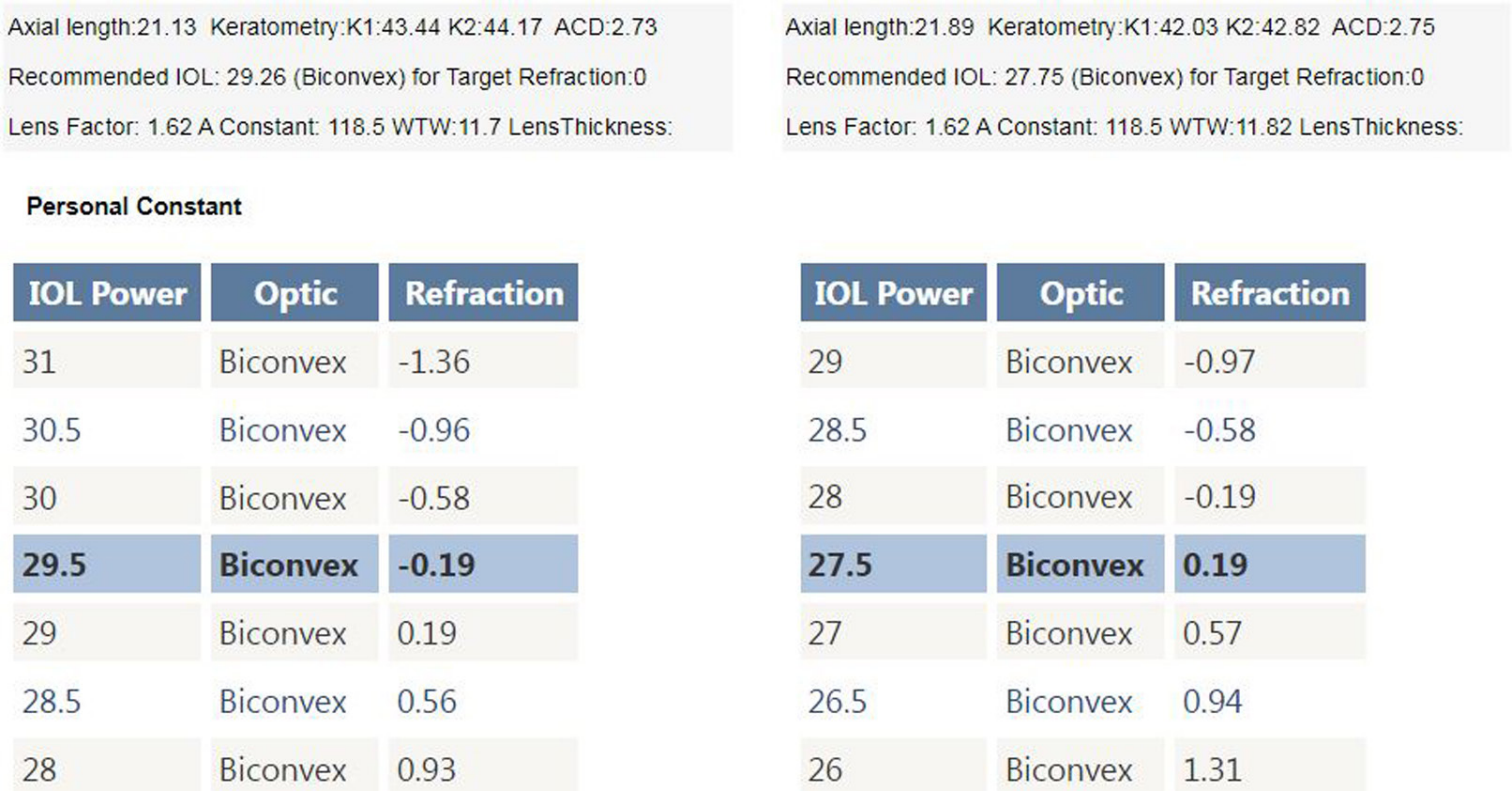
IOL power calculation printout with Barrett Universal II for HOYA iSert 250 (https://calc.apacrs.org/barrett_universal2105).

After discovering that the reason for the unexpected refraction was due to the incorrect power of the IOL, which was caused by a mislabeling from the manufacturer, we decided to proceed with an IOL exchange surgery. The patient was consulted and agreed to undergo the procedure. We recalculated the IOL power using the Barrett Universal II formula and a 118.5 A constant (Figure 3), which confirmed that the proper power was +29.5D. So, we confidently used the exact biometric measurements from the original biometry. However, we decided to use a different brand of IOL at the patient's request and chose a+29.5D Hoya iSert® 250 monofocal hydrophobic acrylic one‐piece IOL. The surgery was successfully performed without any complications, and the IOL was removed without any injury to its optics using a twist‐and‐out technique.

Five days after the surgery, we decided to check the patient's refraction due to her history of refractive surprise. The results showed that her right eye had a refraction of +0.5–1 * 180 with a BCVA of 20/20. Her vision remained stable during her visits 1 and 3 months later. We attempted to measure the first IOL power in vivo using manual and automated lens meters but were unsuccessful. As a result, we contacted both the manufacturer and supplier companies for assistance with checking the IOL power. However, we are still waiting to receive a response from either company.

## 5 DISCUSSION

After cataract surgery, experiencing a refractive surprise is an unpleasant issue for both patients and surgeons. There could be multiple reasons for this problem, and each possibility should be thoroughly investigated. One of the rare causes of this issue is the mislabeling of the IOL. So far, only three cases of IOL mislabeling have been reported.^3^, ^4^, ^5^

Incorrect biometry readings, such as measurements of axial length or keratometry, are common reasons for inaccurate calculation of IOL power. To manage postoperative refractive surprise effectively, it is essential to prevent it in the first place.

Surgical confusion, including wrong patient, wrong site, wrong eye surgery, and incorrect implant placement, can also lead to refractive surprises in cases of wrong IOL implantation.^6^ The adoption of surgical checklists has helped to reduce surgical morbidity and mortality, and these have been widely implemented.^2^

The Pentacam Scheimpflug camera can be used to measure the central optic thickness of an intraocular lens (IOL) and calculate its power. This is done using a regression equation, which provides a 95% confidence that the predicted value will be less than 1.0 D from the true IOL power. However, this method could be improved in its effectiveness for Alcon IOLs.^7^

Measuring the IOL power in situ can be done in another way by comparing the central optic thickness of a different patient with the same IOL type, brand, and power using a Pentacam Scheimpflug camera with that of the patient. However, this method is yet to be confirmed and requires further studies. It is important to note that Pentacam‐based methods have certain limitations that can affect measurements. For instance, measurements can be affected when the patient's IOL is tilted or decentered.

Another useful method for measuring IOL power in situ, is using anterior segment optical coherence tomography. AS‐OCT can measure central IOL thickness with high repeatability and correlation with labeled IOL power. This can predict IOL power with ±0.85 D, which is useful in cases of postoperative IOL surprise.^8^

A precise and effective way to calculate the power of an IOL in place is by using ray tracing. This method involves analyzing the postoperative refraction and biometric measurements to determine the power of the IOL in situ accurately.^9^

Olsen et al. developed a technique for measuring IOL power using a keratometer. The method involves measuring the curvature of the steepest surface of the IOL with a minus lens, which facilitates measuring mires within the keratometer's range.^10^

We were unable to verify the primary IOL power in vivo. However, the fact that we observed an improvement in postoperation refraction after using the same IOL powers with the same A constant and with the involvement of +6D indicates that the manufacturer mislabeled the IOL, which is the cause of the refractive surprise.

There are various methods available to address refractive surprise after cataract surgery. These include corneal‐based surgery and lens‐based procedures, which depend on the extent of the residual error.^11^ If the residual error exceeds the capacity of corneal refractive surgery, then IOL exchange or piggyback IOLs are the following options to consider. However, it is essential to note that lens‐based surgery has multiple risks, such as corneal endothelial decompensation, posterior capsular rent, vitreous loss, and unsatisfactory visual outcomes.^4^

Borgan et al. conducted a comprehensive investigation of cataract surgery refractive outcomes at a large scale, which revealed that 12% of patients experienced a refractive surprise greater than 1 diopter of target refraction.^12^ The underlying cause of these low‐amount refractive surprises remains unknown, and determining the extent to which IOL mislabeling contributes to these outcomes is challenging.

To conclude, the mislabeling of IOLs can be an unpredictable and problematic issue for surgeons, and there is no clear solution to prevent it. However, we can take measures to prevent the implantation of incorrect IOLs due to other reasons by carrying out precise biometry to determine the accurate IOL power and then safely implanting the correct IOL. This case emphasizes the significance of accurate labeling and verification of IOLs to ensure that cataract surgery yields optimal results.

## AUTHOR CONTRIBUTIONS

**Saeed Shokoohi Rad:** Conceptualization; methodology; supervision; validation; visualization. **Mohammad Reza Ansari Astaneh:** Data curation; writing – review and editing. **Mohammad Yaser Kiarudi:** Methodology; project administration; supervision; writing – review and editing. **Seyed Hossein Ghavami Shahri:** Investigation; visualization; writing – original draft. **Hamid Reza Heidarzadeh:** Data curation; methodology; writing – original draft; writing – review and editing.

## FUNDING INFORMATION

The authors received no funding. It is the author's own work, not funded by the government or academic institutes.

## CONFLICT OF INTEREST STATEMENT

The authors declare that they have no competing interests.

## CONSENT

Written informed consent was obtained from the patient to publish this report in accordance with the journal's patient consent policy.

## Data Availability

The datasets used during the current study are available from the corresponding author upon reasonable request.
